# Ribonuclease zymogen induces cytotoxicity upon HIV-1 infection

**DOI:** 10.1186/s12981-021-00399-z

**Published:** 2021-10-26

**Authors:** Ian W. Windsor, Dawn M. Dudley, David H. O’Connor, Ronald T. Raines

**Affiliations:** 1 Department of Chemistry, Massachusetts Institute of Technology, Cambridge, MA 02139 USA; 2 Department of Biochemistry, University of Wisconsin−Madison, Madison, WI 53706 USA; 3 Department of Pathology and Laboratory Medicine, University of Wisconsin−Madison, Madison, WI 53706 USA; 4 Department of Chemistry, University of Wisconsin−Madison, Madison, WI 53706 USA; 5 Koch Institute for Integrative Cancer Research at MIT, Massachusetts Institute of Technology, Cambridge, MA 02142 USA; 6 Present Address: Laboratory of Molecular Medicine, Boston Children’s Hospital, Harvard Medical School, Boston, MA 02115 USA

## Abstract

**Background:**

Targeting RNA is a promising yet underdeveloped modality for the selective killing of cells infected with HIV-1. The secretory ribonucleases (RNases) found in vertebrates have cytotoxic ribonucleolytic activity that is kept in check by a cytosolic ribonuclease inhibitor protein, RI.

**Methods:**

We engineered amino acid substitutions that enable human RNase 1 to evade RI upon its cyclization into a zymogen that is activated by the HIV-1 protease. In effect, the zymogen has an HIV-1 protease cleavage site between the termini of the wild-type enzyme, thereby positioning a cleavable linker over the active site that blocks access to a substrate.

**Results:**

The amino acid substitutions in RNase 1 diminish its affinity for RI by 10^6^-fold and confer high toxicity for T-cell leukemia cells. Pretreating these cells with the zymogen leads to a substantial drop in their viability upon HIV-1 infection, indicating specific toxicity toward infected cells.

**Conclusions:**

These data demonstrate the utility of ribonuclease zymogens as biologic prodrugs.

**Supplementary Information:**

The online version contains supplementary material available at 10.1186/s12981-021-00399-z.

## Introduction

Numerous HIV antiviral compounds effectively target multiple aspects of the viral lifecycle [1]. These compounds, in combination, can suppress the viral load of patients to below detectable limits. These treatments have transformed HIV from a death sentence to a chronic illness. Still, treatment must be continuous, as viremia rebounds upon suspension of therapy [2], and a cure remains elusive [3].

The key challenge to HIV eradication is the immediate establishment of a reservoir of latently infected cells that harbor the integrated HIV provirus without producing viral RNA or proteins [4]. One approach is to stimulate virus production to induce cytopathic effects, but viral activation alone is insufficient to kill latently infected cells [5]. An alternative, or perhaps complementary, strategy is to target cells that produce viral proteins with cytotoxic therapeutics [6].

Secretory ribonucleases (RNases) from vertebrates provide an attractive platform to engineer therapeutics that engender cytotoxicity specifically to HIV-infected cells. In roles ranging from cell signaling to innate immunity, endogenous RNases enter cells and cleave cytosolic and nuclear RNA [7]. Several human RNases are innate immune factors that limit HIV replication in vitro [8, 9]. The cytotoxic ribonucleolytic activity of RNases is kept in check by the cytosolic ribonuclease inhibitor protein, RI. This anionic, horseshoe-shaped protein binds RNases with sub-femtomolar affinities—the highest known for protein–protein interactions [10, 11]. We have reported the design of RNase variants that evade RI and exert cytotoxicity in cancer cells [12]. Further, we have developed circularly permuted zymogens (that is, inactive precursors) of bovine RNase A and more recently, circular zymogens of human RNase 1 that are activated by disease-related proteases [13–16]. Here, we combine these strategies to create a ribonuclease zymogen that is activated within human cells upon HIV-1 infection, providing a targeted approach to disrupt the production of infectious HIV viral particles.

## Materials and Methods

### Protein expression and purification

QBI-139, which is an RI-evasive variant of human RNase 1 that is undergoing clinical trials as a cancer chemotherapeutic agent [17, 18], was obtained from Quintessence Biosciences (Madison, WI).

Like QBI-139, the ribonuclease zymogen in this work was derived from human RNase 1 (Additional file 1: Figure S1). The zymogen relied on five substitutions in QBI-139 (G38R, R39G, N67R, G89R, and S90R) that increase its cytotoxicity by lowering its affinity for RI. These substitutions thereby enable the hydrolysis of cytosolic RNA while not compromising cellular internalization, which is mediated by cationic side chains interacting with anionic membranes. The substitutions were installed in a cyclic variant of RNase 1 in which the N and C termini are linked by the optimized HIV-1 protease cleavage site SGIFLETS [19, 20]. A gene that directs the expression of the zymogen, which we refer to as “Str2-EV”, was generated by encoding the *Nostoc punctiform* (Npu) DnaE intein fragments in the pET32b *Escherichia coli* expression vector, as described previously [16]. In Str2-EV, “Str” refers to “strain”, resulting from four and six residues being excised from the original N and C termini, respectively [16]; and “EV” refers to the “evasion” of RI from the five substitutions of QBI-139. Synthetic DNA for the production of the gene encoding Str2-EV was obtained from Integrated DNA Technologies (Coralville, IA).

Str2-EV was produced in its cyclic form in cells of *E. coli* strain DE3 and purified by methods described previously [16]. Str2-EV precipitates when stored at high concentrations (20 mg/mL) at 4 °C, and was instead flash-frozen in N_2_(l) for storage at −80 °C. Soluble protein was recovered by thawing rapidly at 37 °C. Human RI [21] and pseudo-wild-type HIV-1 protease (which was the Q7K/L33I/L63I/C67A/C95A variant [20]) were produced and purified as described previously.

### Enzymatic activity assays

Ribonucleolytic activity was monitored by using the fluorogenic substrate 6-FAM–dArUdAdA–6-TAMRA [22]. Initial (*I*_o_) and final (*I*_f_) intensities along with linear slopes (Δ*I*/Δ*t*) or the second derivative of quadratic fits (Δ^2^*I*/Δ*t*^2^) were measured and used to calculate values of *k*_cat_/*K*_M_, as described previously [22]. Fluorescence intensity was measured with an M1000 microplate reader from Tecan (Männedorf, Switzerland). Assays were performed in quadruplicate in a flat, black 96-well plate from Corning (Corning, NY). Assay buffers were treated with diethyl pyrocarbonate, which inactivates contaminating ribonucleases [23], and consisted of either 50 mM Tris–HCl buffer, pH 7.4, containing NaCl (100 mM) and substrate (20 nM), or 50 mM sodium acetate buffer, pH 5.0, containing NaCl (100 mM), substrate (20 nM), and HIV-1 protease (50 nM).

The *k*_cat_/*K*_M_ value for ribonucleolytic catalysis was calculated with Eq. 1.

1 $$ \frac{{k_{{{\text{cat}}}} }}{{K_{{\text{M}}} }} = \frac{{{\raise0.7ex\hbox{${\Delta I}$} \!\mathord{\left/ {\vphantom {{\Delta I} {\Delta t}}}\right.\kern-\nulldelimiterspace} \!\lower0.7ex\hbox{${\Delta t}$}}}}{{\left[ {{\text{RNase}}} \right]\left( {I_{{\text{f}}} - I_{{\text{o}}} } \right)}} $$

The *k*_cat_/*K*_M_ value for zymogen cleavage by HIV-1 protease was calculated with Eq. 2 by assaying the increase in ribonucleolytic activity upon the addition of HIV-1 protease, as described previously [16].

2 $$ \frac{{k_{{{\text{cat}}}} }}{{K_{{\text{M}}} }}_{{{\text{protease}}}} = \frac{{{\raise0.7ex\hbox{${\Delta^{2} I}$} \!\mathord{\left/ {\vphantom {{\Delta^{2} I} {\Delta t^{2} }}}\right.\kern-\nulldelimiterspace} \!\lower0.7ex\hbox{${\Delta t^{2} }$}}}}{{\left( {\frac{{k_{{{\text{cat}}}} }}{{K_{{\text{M}}} }}_{{\text{activated zymogen}}} - \frac{{k_{{{\text{cat}}}} }}{{K_{{\text{M}}} }}_{{{\text{zymogen}}}} } \right)\left[ {{\text{zymogen}}} \right]\left[ {{\text{protease}}} \right]\left( {I_{{\text{f}}} - I_{{\text{o}}} } \right)}} $$

Values of *k*_cat_/*K*_M_ at pH 7.4, which is near the optimal pH for catalysis by RNase 1 [24], are reported in the main text. These values were also determined at pH 5.0, which is near the optimal pH for catalysis by HIV-1 protease [25], and used as parameters in fitting Eq. 2 (Additional file 1: Table S1).

The ability of proteases in a cell extract to cleave a peptide that corresponds to the linker within the zymogen was assessed by using the fluorogenic substrate RE(EDANS)SGIFLETSK(DABCYL)R obtained from Biomatik USA (Wilmington, DE) [20]. Cell extract was prepared by pelleting 2  ×  10^6^ MT-4 cells and resuspending them in 200 μL of M-PER Mammalian Protein Extraction Reagent from ThermoFisher Scientific (Waltham, MA). Lysis was conducted on a shaker for 10 min followed by clarification by centrifugation at 14,000*g* for 15 min. The protein concentration of the cell extract was determined with the Pierce™ BCA Protein Assay Kit from ThermoFisher Scientific (Waltham, MA). A stock of 1.0 mg/mL extracted protein was prepared by dilution with M-PER along with a two-fold dilution series and used immediately without the addition of protease inhibitors. Peptide was added to the wells of a 96-well plate to a final concentration of 10 μM in PBS followed by varying concentrations of cell extract (20-fold dilution of M-PER stocks). Fluorescence intensity was measured over time in triplicate for each cell extract concentration to obtain the initial velocity in RFU/s, which was converted to nM/s with a product standard curve (70 RFU per nM product [20]). The slope of product formation in nM/s plotted against mass of cell extract (Additional file 1: Figure S4), 3.3 nM/s/mg, was used to compare against the previously reported initial velocity of 0.68 nM/s for the turnover of 10 μM substrate by 214 pM HIV protease (21.7 kDa homodimer) in 200 μL (7.3  ×  10^5^ nM/s/mg) [20].

### Protein thermostability assays

Protein thermostability was determined by differential scanning fluorometry. Thermal denaturation experiments were conducted in 20 µL of PBS containing a ribonuclease (30 µg) and SYPRO Orange (0.6% w/v). Denaturation data were obtained with a Quant studio 7 RT PCR machine from Applied Biosystems (Foster City, CA) by increasing the temperature from 20 to 96 °C at 1 °C/min. The value of *T*_m_, which is the temperature at the midpoint of the thermal transition from the native to the unfolded state, was determined by the Boltzmann model using Protein Thermal Shift software from Applied Biosystems.

### Enzyme inhibition assays

The inhibition of ribonucleases was assessed by measuring the change in the initial reaction rate using the activity assay described above. QBI-139 is more active than proteolytically activated Str2-EV, and concentrations as low as 35 pM could afford useful initial rates. This enzyme concentration was well below the IC_50_ value for the inhibition of QBI-139 by RI. Thus, as the substrate concentration was  ≪ *K*_M_, *K*_i_ ≈ IC_50_ [26]. Nonlinear fitting of the log[RI] as a function of raw initial rates (RFU/s) was performed with the asymmetric sigmoidal 5-parameter logistic equation of Prism 7 software from Graphpad (San Diego, CA) (Additional file 1: Figure S2). The *K*_M_  =  22 µM of a related nonanucleotide substrate that is cleaved with similar efficacy was employed as a constrained fitting parameter along with the fixed enzyme and substrate concentrations employed in the activity assay. The ensuing values of *K*_i_ are listed in Table 1.

**Table 1:** Biochemical properties of ribonucleases and their zymogens

Ribonuclease or peptide	*k*_cat_/*K*_M_ (inactive) (M^−1^ s^−1^)	*k*_cat_/*K*_M_ (activated) (M^−1^ s^−1^)	*k*_cat_/*K*_M_ (relative)	*T*_m_ (°C)	*k*_cat_/*K*_M_ (HIV-1 protease) (M^−1^ s^−1^)	*K*_i_ by RI (M)
RNase 1 [21]	NA	2.1 × 10^7^	NA	57	NA	2.9 × 10^−16^
QBI-139	NA	4.3 × 10^5^	NA	78 ± 2	NA	6 ± 1 × 10^−10^
SGIFLETS [20]	NA	NA	NA	NA	5.0 × 10^5^	NA
Str2^a^ [16]	5.8 × 10^3^	6.7 × 10^7^	11,000	47.5	3.9 × 10^3^	ND
Str2-EV^a^	2.0 × 10^2^	8.5 × 10^3^	42	50.0 ± 0.1	4.1 × 10^3^	7 ± 1 × 10^−10^

*NA* not applicable; *ND* not determined

^a^Values of *T*_m_ and *K*_i_ were determined with activated zymogen

Str2-EV exhibited low catalytic efficiency. We were unable to measure an IC_50_ that was higher than the enzyme concentration employed, which necessitated the use of Morrison’s equation for tight-binding inhibitors. Concentrations were devised to sample the three critical regions of the curve efficiently [27]. Raw initial rates (RFU/s) as a function of RI concentration were fitted by non-linear regression analysis with the “Morrison *K*_i_” equation in Prism 7 software (Additional file 1: Figure S2). Values of *K*_i_ and the standard error derived from this fit are listed in Table 1.

### Cell and virus cultures

MT-4 cells [28] and a plasmid encoding the NL4-3 virus [29] were obtained from the NIH AIDS reagent program (Items 120 and 114, respectively). Cell densities were measured with a Countess cell counter from Thermo Fisher Scientific (Waltham, MA) using bright-field imaging and exclusion staining with trypan blue from Invitrogen (Carlsbad, CA). MT-4 cells were maintained at 0.2–1.0  ×  10^6^ cells per mL in RPMI 1640 medium containing heat-inactivated FBS (10% v/v) and HyClone antibiotic–antimycotic solution from GE Healthcare (Chicago, IL) (1% v/v) at 37 °C in a CO_2_ (5% v/v) atmosphere. MT-4 cells, 1 mL at a density of 10^6^ cells/mL, were transfected with pNL4-3 plasmid (5 µg) using the Xfect reagent from Clontech (Mountain View, CA). Supernatants were harvested on Day 6 and frozen for use in subsequent infections. The titer (TCID_50_) of viral stocks was determined by the proportionate distance method [30].

Experimental cell culture was conducted in tissue culture-treated, flat-bottom, 96-well microplates, which were product 3595 from Corning, starting with 2.0  ×  10^5^ cells/mL in 200 µL of medium containing PBS (10% v/v). Experimental infections were conducted by resuspending pelleted cells at 10^8^ cells/mL and infecting with 100 TCID_50_s of Day 6 supernatants by spinoculation for 2 h at 1200 rcf followed by a 2-h incubation at 37 °C with 5% CO_2_. Infected cell pellets were washed by dilution to 2.0  ×  10^5^ cells/mL and subsequent pelleting and resuspension at 4.0  ×  10^5^ cells/mL for use as a 2×  stock for plating. In Str2-EV pretreatment studies, 10 mL of 2.5  ×  10^5^ cells/mL were grown for 24 h in the presence of zymogen (5 µM). After the incubation and prior to infection, cells were again counted by trypan exclusion staining to account for growth and were infected and plated as described above.

### Cell viability assays

Cell viability was assessed by using the CellTiter 96^®^ AQ_ueous_ One Solution Cell Proliferation MTS Assay from Promega (Madison, WI), which is based on the reduction of a tetrazolium dye in living cells [31]. Assays were conducted by adding 20 µL of the MTS reagent to 100 µL of cells harvested from experimental 96-well plates. Absorbance was measured at 492 nm after a 2-h incubation at 37 °C.

### ELISAs

The p24 content of HIV-infected cell culture supernatants was measured with the Lenti-X™ p24 Rapid Titer Kit from Takara Bio (Mountainview, CA). Supernatants from each control condition representing the highest expected virus content were removed (5 µL) and diluted to empirically determine the optimal dilution factor for a given experiment to enable measurements in the linear range of the assay (12.5–200 pg/mL), which typically required 10^3^- to 10^4^-fold dilutions. Absorbance was measured at 450 nm. Values are recorded as the concentration and percentages of an untreated control.

## Results and discussion

### In vitro characterization of the RI-evasive RNase, QBI-139

Cellular internalization, evasion of RI, and efficient ribonucleolytic activity are the key determinants of secretory ribonuclease cytotoxicity [7]. Initial efforts to evade RI focused on RNase A and revealed that a substitution (G88R) deep in the RI–RNase A interface increased the cytotoxicity of RNase A by reducing its affinity for RI [32]. Translating these results to the human homolog RNase 1 required more extensive alteration to achieve RI evasion [21, 33]. Early attempts sought to replace cationic residues at the RI-binding interface with anionic residues to disrupt charge-mediated interactions [21]. This approach did diminish the affinity for RI but also reduced cellular internalization because cationic residues are essential for Coulombic interactions with anionic membrane-anchored components on the cell surface in the initial step of the internalization pathway [34]. Additional studies revealed reducing RNase 1 cationicity was correlated with a reduction in RI affinity as well as RNase internalization [21]. Further elaboration identified arginine substitutions that imparts a large positive formal charge to both maintain cellular uptake and disrupt RI binding, imbuing a variant of RNase 1, QBI-139, with clinically useful cytotoxicity [17, 18].

We began assessing the suitability of QBI-139 as a cytotoxin for antiretroviral therapy by characterizing desirable in vitro properties: ribonucleolytic activity, thermostability, and RI affinity. Using a fluorogenic substrate, we found that the catalytic efficiency of QBI-139 is two orders-of-magnitude lower than that of wild-type RNase 1 (Table 1). The extraordinary affinity of RI toward wild-type RNases requires measuring association and dissociation rate constants to calculate the equilibrium dissociation constant. In contrast, the significantly weaker affinity of RI towards QBI-139 enabled us to perform conventional inhibition kinetics. Titrating QBI-139 with RI revealed the enzyme concentration employed in the assay was lower than the IC_50_ and thus approximates the inhibition constant (*K*_i_) (Table 1, Additional file 1: Figure S2). The *K*_i_  =  0.6 nM of QBI-139 is a million-fold greater than the *K*_d_ value of the complex of RI with wild-type RNase 1. Finally, we used differential scanning fluorometry to measure the thermostability of QBI-139, which exhibits a 21 °C increase in *T*_m_ relative to RNase 1 (Table 1).

### Design and characterization of the RI-evasive zymogen, Str2-EV

**Fig. 1:**
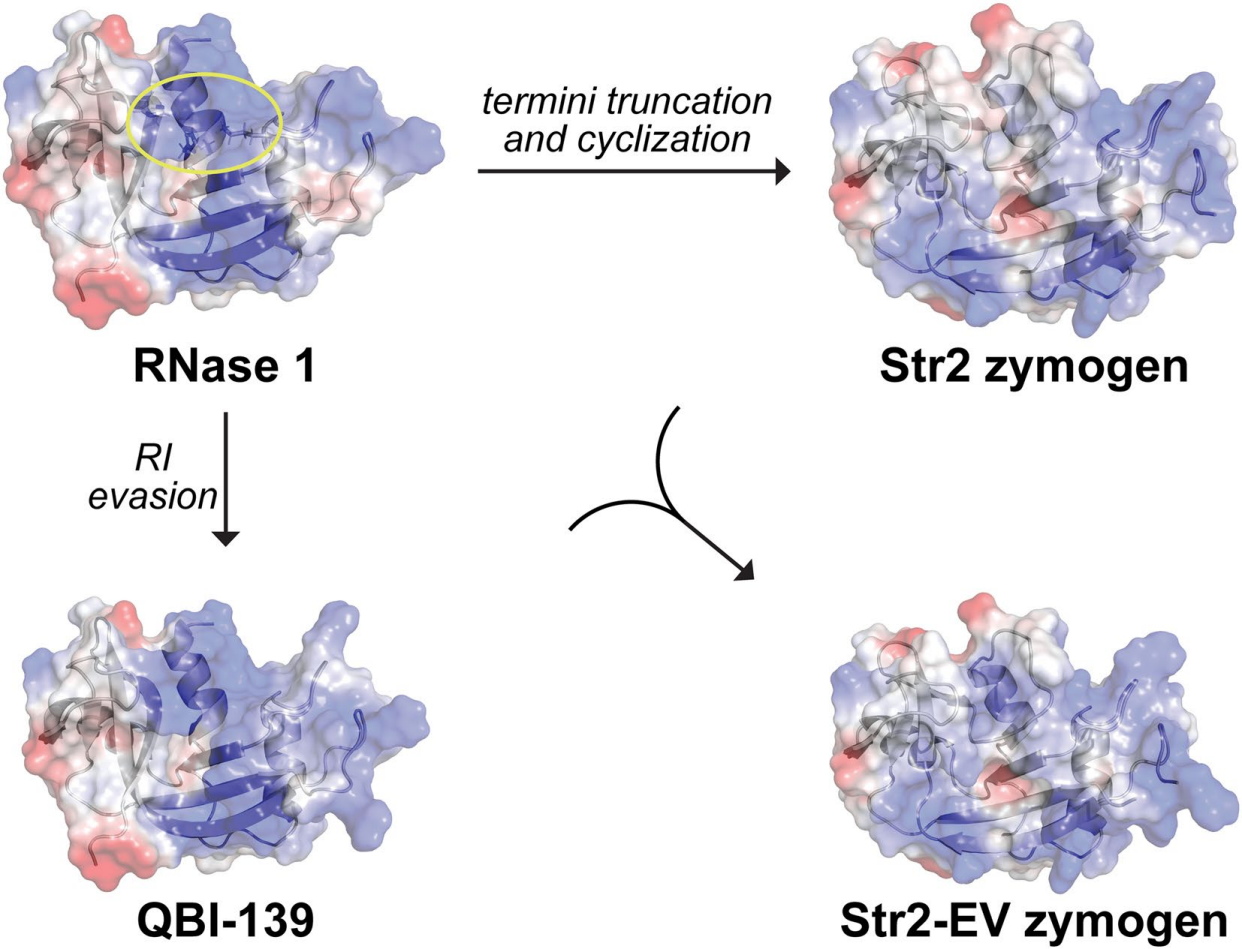
Electrostatic potential maps of ribonucleases and their zymogens. Wild-type human RNase 1 has a cationic active-site (yellow oval). Cyclization to form a zymogen blocks that active site. QBI-139 has cationic residues grafted at the RI-binding interface. The image of RNase 1 is from PDB entry 1z7x [21]. A model of Str2 was built with Rosetta as described previously [16]. Models of QBI-139 and Str2-EV were built with PyMOL 2.0.4 software, which was also used to generate the electrostatic surface maps

Previously, we reported on circular zymogens of wild-type RNase 1 in which regulatory control is imposed by installing a protease-cleavage site between the native termini using intein-mediated cis-splicing [16]. The termini are located at the ends of the active-site cleft. The truncation of inconsequential residues at the termini, residues 1–4 and 123–128, brought the linker closer to the active site and imposed strain, leading to 10^4^-fold inactivation. This variant, Str2, is also a substrate of HIV-1 protease, which restores wild-type activity (Table 1). Installation of the linker obscures the cationic active-site cleft and obfuscates interactions that are important for cellular internalization (Fig. 1). We therefore installed substitutions derived from QBI-139 (G38R, R39G, N67R, G89R, and S90R) to create the RI-evasive zymogen Str2-EV.

We characterized the in vitro properties of Str2-EV before embarking on antiviral studies. Like its Str predecessor [16], Str2-EV has low ribonucleolytic activity. HIV-1 protease cleaves Str2-EV with *k*_cat_/*K*_M_  =  4.1 × 10^3^ M^−1^ s^−1^, and the zymogen gains 42-fold in ribonucleolytic activity upon proteolytic activation. The *T*_m_  =  50.0 °C of Str2-EV is slightly greater than that of Str2. Finally, we found the affinity of RI for Str2-EV to be indistinguishable from that of QBI-139 (Table 1, Additional file 1: Figure S2).

### Assessing the antiviral therapeutic window of Str2-EV zymogen

QBI-139 and Str2-EV were evaluated for cytotoxicity and efficacy at suppressing production of viral proteins. Uninfected CD4^+^ cells (MT-4 cell line) were treated with varying amounts of QBI-139 and Str2-EV, and cell viability was assessed after 7 days to determine a 50% inhibitory concentration (IC_50_) (Table 2, Additional file 1: Figure S3). QBI-139 was determined to be 21-fold more toxic than Str2-EV. Next, we measured the ability of QBI-139 and Str2-EV to inhibit viral replication by measuring the amount of p24 produced after 7 days following a post-infection treatment with varying RNase concentrations. The IC_50_ values for viral suppression are only slightly lower than the cytotoxicity (Table 2). These data indicate that, if a therapeutic window exists for Str2-EV, it is narrow.

**Table 2:** Cellular activities of ribonucleases

Ribonuclease	Cell death (IC_50_)^a^	HIV inhibition (IC_50_)^b^
QBI-139	0.41 µM	0.34 µM
Str2-EV	8.7 µM	7.5 µM

^a^Determined after 7 days with a tetrazolium-dye based assay or

^b^p24 ELISA

### Assessing pretreatment antiviral properties of Str2-EV

**Fig. 2:**
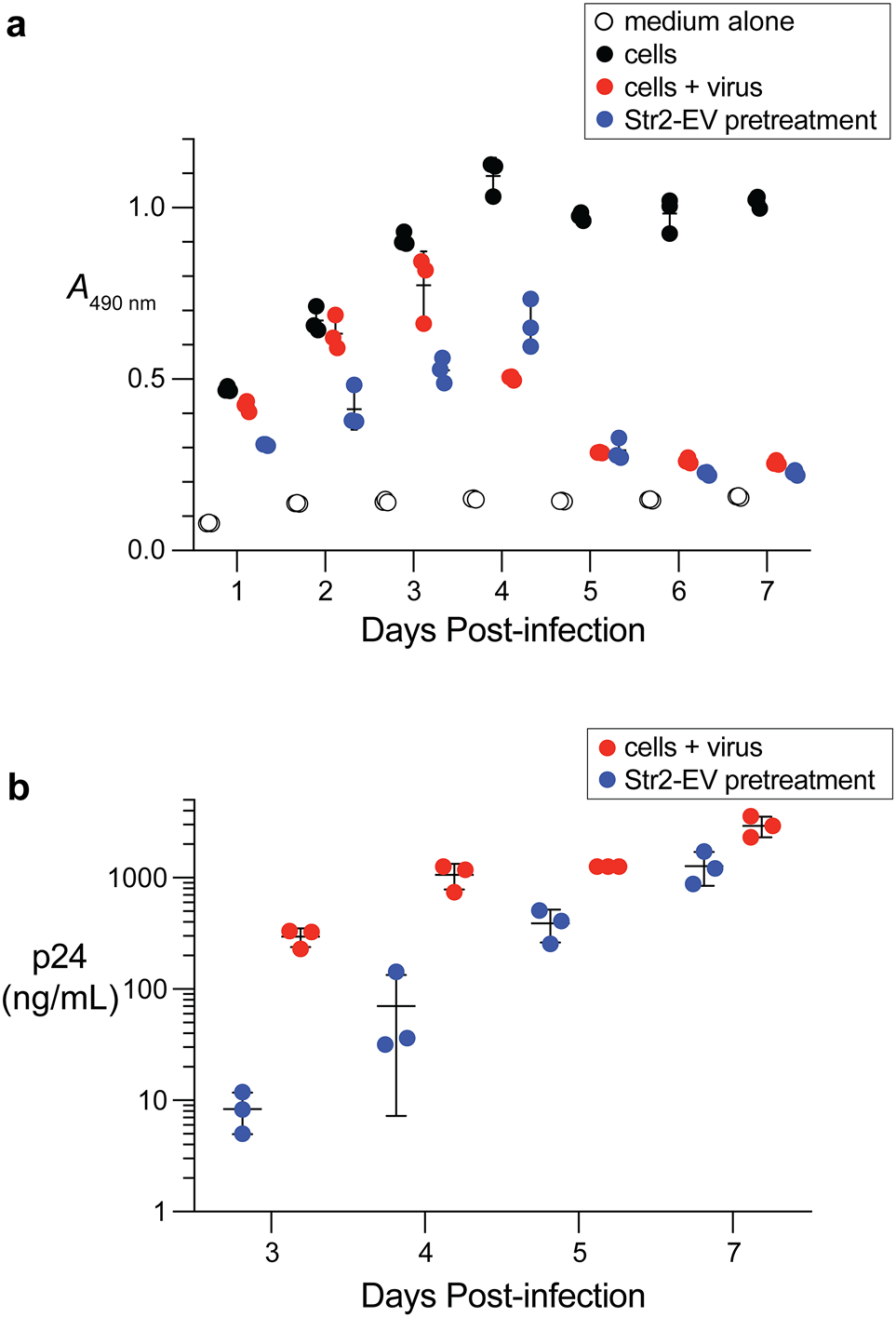
Graphs showing the effect of the Str2-EV zymogen on HIV-induced cell death and viral production. **a** The viability of MT-4 cells was monitored with an tetrazolium dye-based assay to evaluate the effect of viral infection and pretreatment with the Str2-EV zymogen. **b** The production of HIV-1 virus was measured from the samples in panel A with an ELISA for p24, which is a viral capsid protein. All assays were performed in triplicate; data are the mean  ±  SD

Following the unexpectedly high cytotoxicity of the Str2-EV zymogen, we investigated whether cells pretreated with Str2-EV exhibited specific cytotoxicity upon HIV infection. Cells were incubated with 5 µM, which is the highest concentration of Str2-EV that did not exhibit any reduction in viability (Additional file 1: Figure S3), for 24 h prior to infection. A large drop in viable cells (42%) was observed on the first day post-infection for infected cells pretreated with the zymogen compared to a modest drop (13%) resulting from HIV-1 infection (Fig. 2). Following the reduction in viable cells, the production of viral proteins is diminished by greater than an order of magnitude at days 3–5 post-infection. Eventually, on day 7 post-infection, the viability of treated cells drops to that of the infected control. These results indicate that those cells that had internalized the Str2-EV died upon infection with HIV-1.

### Assessing non-specific activation of linker peptide

The high cytotoxicity of the Str2-EV zymogen prompted us to investigate the potential for its activation in cellulo in the absence of HIV-1 protease by monitoring the cleavage of a peptide containing the inactivating zymogen linker (SGILETS). Cell extract hydrolyzed a fluorogenic peptide with a specific activity of 3.3 nM/s per mg of extracted protein (Additional file 1: Figure S4). Purified HIV-1 protease cleaves that same peptide with a specific activity of 7.3  ×  10^5^ nM/s per mg of enzyme, representing a 2  ×  10^5^-fold greater activity on a mass basis. A comparison of the catalytic efficiency of the isolated peptide and the Str2-EV zymogen shows the peptide is cleaved 10^2^-fold more efficiently (Table 1), and we suspect a similar result for non-specific activation by the cell extract of the Str2-EV zymogen.

## Conclusion

Herein, we report on a de novo engineered zymogen, Str2-EV, that has demonstrable antiviral activity in live cells. Though Str2-EV does not have a significant therapeutic window post-infection, pretreatment does lead to rapid cell death in HIV-infected cells. Our findings motivate the continuing development of ribonuclease zymogens, and we anticipate two key features of HIV-1 protease activated zymogens that could be further optimized: non-specific activation and cellular delivery. Peptide hydrolysis by a cell extract reveals the kinetically optimized substrate utilized in the inactivating linker can be cleaved non-specifically, which likely underlies the high cytotoxicity of the Str2-EV zymogen. HIV-1 protease and other retroviral proteases can uniquely cleave the peptide bond before proline residues, and substitution of the P1′ residue provides an avenue to enhance activation specificity. The lack of a therapeutic window and pretreatment requirement for Str2-EV antiviral activity might be due to viral entry being faster than zymogen internalization. Whereas HIV-1 internalization is mediated by direct membrane fusion [35], internalization by cationic proteins such as secretory ribonucleases requires multiple steps and has relatively low efficiency [36]. We are encouraged by the recent discovery that an anionic, RI-evasive variant of RNase 1 can be endowed with cell permeability by the bioreversible esterification of enzymic carboxylate groups [37], and that esterification could benefit ribonuclease zymogens as well.

We envision that HIV-1 protease activated zymogens of RNase 1 could be clinically deployed in a variety of situations. Once non-specific activation and internalization kinetics are optimized, ribonuclease zymogens could be combined with latency-reversing agents to evaluate their efficacy towards eliminating latent viral reservoirs [38]. The mechanism of HIV-1 protease zymogens are compatible with all types of HIV drugs except for protease inhibitors. Because protease inhibitors are typically employed following treatment failure, zymogen ribonucleases could complement standard frontline drugs and pre-exposure prophylaxis regimes. In addition to eradicating viral reservoirs in HIV patients, tissues contaminated with HIV-infected cells could potentially be cleared of infection prior to allo- or autotransplantation through the use of ribonuclease zymogens. The lack of cell specificity is advantageous in reaching all cell types capable of harboring HIV provirus.

## Supplementary Information

**Additional file 1: Table S1.** Str2-EV kinetic parameters at pH 5.0. **Figure S1.** Amino acid sequences of human RNase 1 and the cyclic Str2-EV zymogen. **Figure S2.** Inhibition of QBI-139 and Str2-EV by human RI. **Figure S3.** Inhibition of QBI-139 and Str2-EV by human RI. **Figure S4.** Hydrolysis of SGIFLETS peptide by MT-4 cell extract.

## Data Availability

All data generated or analyzed during this study are included in this published article and its additional file.
