# Supplementary material for: Ribonuclease zymogen induces cytotoxicity upon HIV-1 infection
Source: AIDS Res Ther. 2021 Oct 26;18:77. doi: 10.1186/s12981-021-00399-z (PMC8549155; doi:10.1186/s12981-021-00399-z)
Supplement: Supplementary file 1 — Additional file 1: Table S1. Str2-EV kinetic parameters at pH 5.0. Figure S1. Amino acid sequences of human RNase 1 and the cyclic Str2-EV zymogen. Figure S2. Inhibition of QBI-139 and Str2-EV by human RI. Figure S3. Inhibition of QBI-139 and Str2-EV by human RI. Figure S4. Hydrolysis of SGIFLETS peptide by MT-4 cell extract. [file 12981_2021_399_MOESM1_ESM.pdf]

Ribonuclease Zymogen Induces Cytotoxicity upon HIV-1 Infection

Ian W. Windsor, Dawn M. Dudley, David H. O'Connor, and Ronald T. Raines*

*Correspondence: rtraines@mit.edu

Content	Page
Table of Contents	S1
Table S1 Str2-EV kinetic parameters at pH 5.0	S2
Fig. S1 Amino acid sequences of human RNase 1 and the cyclic Str2-EV zymogen	S2
Fig. S2 Inhibition of QBI-139 and Str2-EV by human RI	S3
Fig. S3 Inhibition of QBI-139 and Str2-EV by human RI	S4
Fig. S4 Hydrolysis of SGIFLETS peptide by MT-4 cell extract	S5

Table S1 Str2-EV kinetic parameters at pH 5.0

k_{cat}/K_M ($M^{-1}s^{-1}$) Inactive	k_{cat}/K_M ($M^{-1}s^{-1}$) Activated
$1.6 \pm 0.1 \times 10^2$	$3.9 \pm 0.2 \times 10^3$

Human RNase 1

```
KESRA KKFQR QHMDS DSSPS SSSTY CNQMM RRRNM TQGRC KPVNT FVHEP
LVDVQ NVCFQ EKVTC KNGQG NCYKS NSSMH ITDCR LTNGS RYPNC AYRTS
PKERH IIVAC EGSPY VPVHF DASVE DST
```

Str2-EV

```
-----A KKFQR QHMDS DSSPS SSSTY CNQMM RRRNM TQRGC KPVNT FVHEP
LVDVQ NVCFQ EKVTC KRGQG NCYKS NSSMH ITDCR LTNRR RYPNC AYRTS
PKERH IIVAC EGSPY VPVHF DASGI FLETS
```

Fig. S1 Amino acid sequences of human RNase 1 and the cyclic Str2-EV zymogen. In the zymogen, substitutions to evade RI are shown in red, the HIV-1 protease recognition site is shown in green, and the linkage created by the *Noctoc punctiforme* DnaE split intein is underlined.

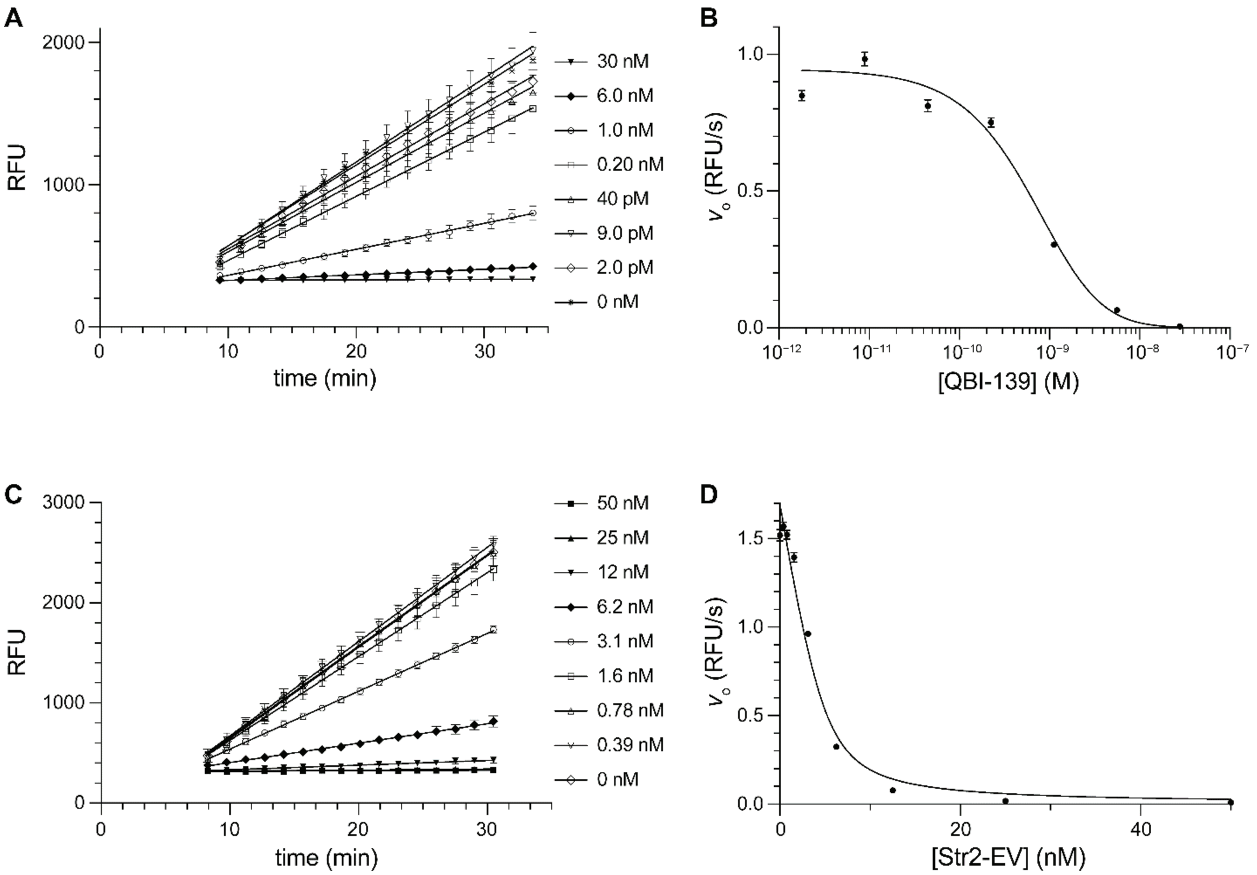

Fig. S2 Inhibition of QBI-139 and Str2-EV by human RI. A. QBI-139 (35 pM) was titrated with RI, and initial velocities were determined by linear regression. B. Initial velocities were plotted on a log scale, enabling the approximation of the K_i using the 5-parameter logistic equation to determine the value of IC_{50}. C. Str2-EV (5 nM) was titrated with RI, and initial velocities were determined by linear regression. D. The ensuing initial velocities were fitted with Morrison's equation for tight binding inhibitors to determine the value of K_i. Values of K_i for QBI-139 and Str2-EV are reported in Table 1.

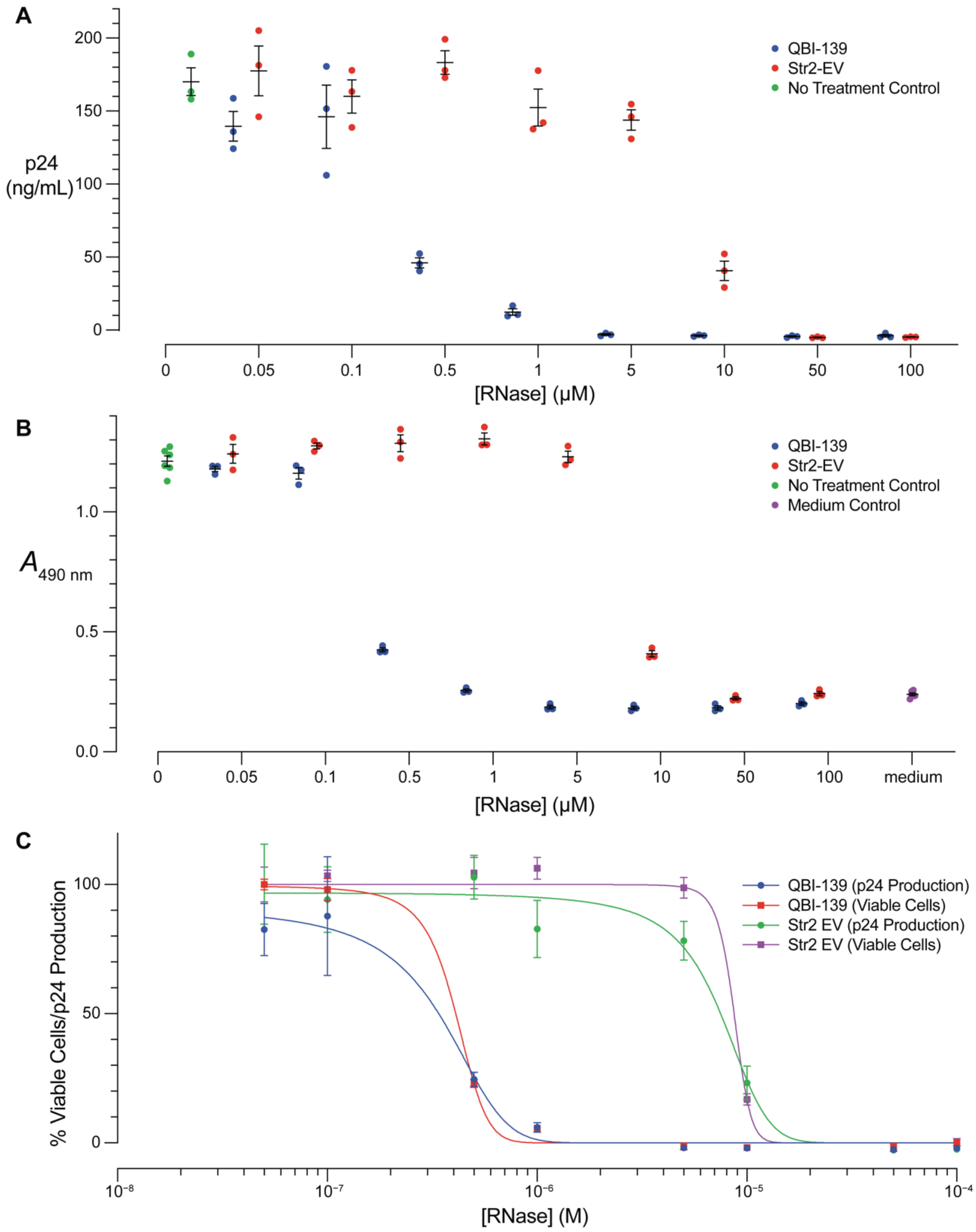

Fig. S3 Concentration-dependence of HIV-1 virus production (A) and cytotoxicity (B). MT-4 cells with (A) or without (B) virus were treated with QBI-139 and Str2-EV. (C) Data from these studies were normalized and fitted with the 5-parameter logistic equation to yield IC_{50} values, which are reported in Table 2.

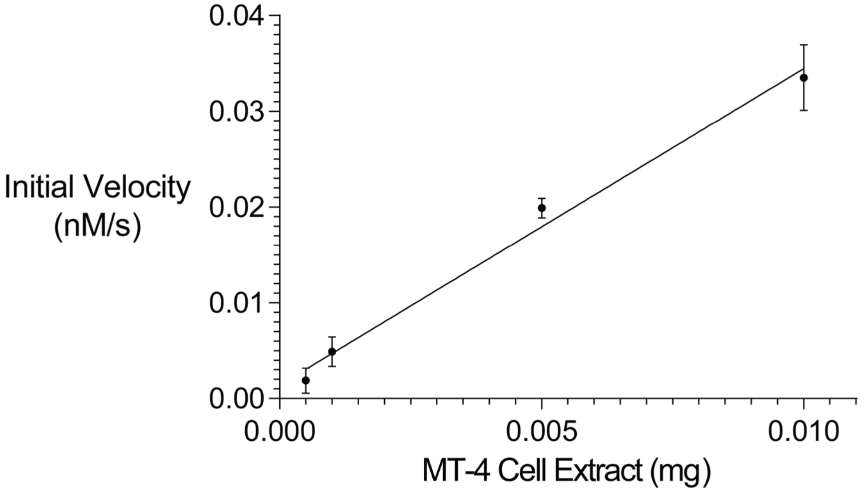

Fig. S4. Hydrolysis of SGIFLETS peptide by MT-4 cell extract. Initial velocities of 10 μM RE(EDANS)SGIFLETSK(DABCYL)R substrate turnover are plotted against the mass of MT-4 cell extract. The slope of the linear least-squares fit of these data is 3.3 ± 0.2 (nM/s)/mg.
